# Physician-led interprofessional pre-hospital teams: does the science hold up?

**DOI:** 10.1186/s13049-025-01368-5

**Published:** 2025-03-27

**Authors:** Ryan Glendwyr Davis, Pieter Francsois Fouche, Belinda Flanagan

**Affiliations:** School of Paramedicine, University of Tasmania, Lilyfield, NSW Australia

Dear Editor,

We read with interest the systematic review and meta-analysis by Lavery et al. evaluating the targeted deployment of physician-led interprofessional prehospital teams for critically ill and injured patients [1]. While this review addresses an important clinical question, we have significant concerns about the robustness of its conclusions due to reliance on observational studies, inadequate adjustment for confounding factors, and misleading causal language.

Firstly, the review predominantly included observational studies, with only one randomised controlled trial (RCT) by Garner et al. [2]. Crucially, Garner et al. demonstrated no significant mortality benefit using intention-to-treat (ITT) analysis. This is important, as ITT analysis preserves randomisation and reduces bias potential when compared to As-Treated (AT) analysis. Lavery et al. conveyed a degree of certainty about the casual benefits of physician-led teams that possibly overstated their findings. Observational studies inherently carry substantial risks of bias and confounding, making causal conclusions inappropriate. We acknowledge that Lavery et al. partially addressed this limitation in their discussion.

Secondly, several included studies compared physician-staffed helicopter emergency medical services (HEMS) directly against ground-based EMS staffed by paramedics, introducing major confounding by differences in transport modality, equipment availability, response times, and crew composition. Galvagno et al. highlighted significant methodological issues and confounding effects inherent in comparisons involving HEMS versus ground EMS, emphasizing the difficulty in isolating the true impact of physician involvement alone [3]. The authors noted this potential confounding as a limitation, though the severity of its implications for their conclusions was understated.

Thirdly, injury severity was inconsistently measured or adjusted for, despite its critical importance in determining outcomes. Only nine studies (39%) reported injury severity scores (ISS), with few using validated physiological scores such as the Trauma and Injury Severity Score (TRISS), Revised Trauma Score (RTS), or Shock Index. Tracey et al. argue for the essential role of these adjustments in critical care research to ensure accurate outcome evaluation [4]. The limited use of such adjustments in Lavery et al.‘s study represents a substantial methodological concern.

Additionally, Lavery et al. reported significant statistical heterogeneity (I² = 73%), indicating substantial variability across studies regarding populations, interventions, and outcomes. Such heterogeneity limits the generalizability and applicability of their pooled results and weakens confidence in their conclusions for guiding policy or clinical practice. While this heterogeneity was recognized as a limitation by Lavery et al., its implication for clinical decision-making requires clearer emphasis.

Lastly, Lavery et al. consistently employed causal language throughout their discussion and conclusions. Given the predominantly observational evidence base, this approach risks overstating findings, potentially misleading clinicians, policymakers, and guideline developers. Del Junco et al. explicitly underscores the importance of cautious interpretation of observational prehospital trauma studies, clearly distinguishing association from causation to prevent premature or misguided clinical and policy decisions [5].

Future research should employ rigorous methodologies, including randomised controlled trials or carefully matched observational studies comparing physician-led and similarly skilled non-physician teams under comparable conditions. Comprehensive adjustment for confounders such as injury severity, comorbidities, patient demographics, transport modality, and response timing is essential. These methodological improvements would yield more internally valid and reliable results to inform prehospital care policies.

We hope this letter will lead to clarification of the concerns raised and encourage the authors to specify in detail some of the limitations of the paper to avoid misguided clinical and policy decisions that may occur as a result.

## Data Availability

No datasets were generated or analysed during the current study.
